# A novel technique of voice-sparing cricotracheal resection

**DOI:** 10.1016/j.xjtc.2023.11.005

**Published:** 2023-11-18

**Authors:** Matthias Evermann, Imme Roesner, Veronika Kranebitter, Doris-Maria Denk-Linnert, Johanna Bauer, Thomas Schweiger, Konrad Hoetzenecker

**Affiliations:** a Department of Thoracic Surgery, Medical University of Vienna, Vienna, Austria; b Division of Phoniatrics and Logopedics, Department of Otorhinolaryngology, Medical University of Vienna, Vienna, Austria

**Keywords:** laryngotracheal surgery, laryngotracheal reconstruction, idiopathic subglottic stenosis, cricotracheal resection, laryngotracheal stenosis

## Abstract

**Background:**

Cricotracheal resection (CTR) is considered the standard of care for patients suffering from idiopathic subglottic stenosis (iSGS). Although CTR results in permanent restoration of airway patency, it has a mild to moderate impact on voice quality. Here we propose modifications of the standard CTR technique to make it a voice-preserving procedure.

**Methods:**

Five women with iSGS underwent voice-sparing CTR between January 2022 and January 2023. In this procedure, through several technical adaptations, the function of the cricothyroid joint was preserved. Outcomes of these voice-sparing CTRs were compared to outcomes in patients who underwent standard CTR in our institution. All patients underwent full functional preoperative and postoperative workups, including spirometry, voice measurements, patient self-assessment, and fiberoptic endoscopic evaluation of swallowing.

**Results:**

All 5 patients in the study group suffered from iSGS with high-grade Myer-Cotton III° stenosis (100%); 1 patient had previously undergone endoscopic laser resection. Voice evaluation demonstrated a nearly unchanged fundamental pitch (mean preoperative, 191 ± 73.1 Hz; postoperative, 182 ± 64.2 Hz) and dynamic voice range (preoperative, 24.4 semitones; postoperative, 20.4 semitones). This was in contrast to the control group, in which significantly reduced voice quality was observed.

**Conclusions:**

In selected patients suffering from iSGS, excellent functional results can be obtained with voice-sparing CTR.

**Figure:**
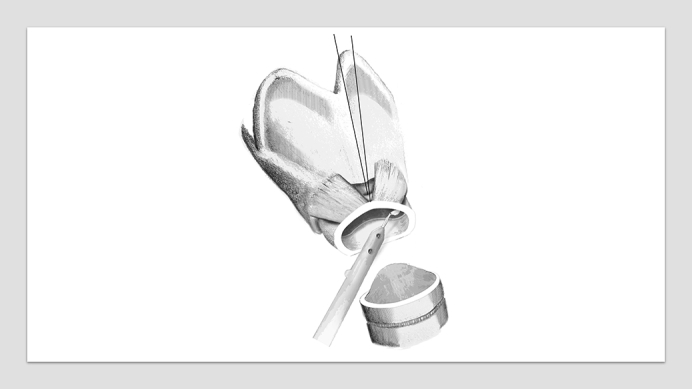
Modified technique for voice-sparing cricotracheal resection.

**Central Message**

Technical modifications to the cricotracheal resection technique have resulted in excellent surgical outcomes with an almost unaltered voice in patients suffering from idiopathic subglottic stenosis.

**Perspective**

Functional impairment of the voice, including a decrease in the fundamental vocal pitch and a significant reduction in dynamic voice range, is well described in patients who undergo cricotracheal resection (CTR). Through several technical refinements, voice quality can be largely maintained after CTR, along with full restoration of respiratory function.

Surgical treatment of idiopathic subglottic stenosis (iSGS) has become a well-established procedure in airway surgery. Excellent long-term results with full restoration of respiratory function have been demonstrated in patients after standard cricotracheal resection (CTR) as well as after extended subglottic resection techniques.^1^, ^2^, ^3^ The main focus of CTR is the complete removal of all affected scar tissue and readaptation of heathy mucosa to healthy mucosa. However, at least a moderate functional impairment of the voice is observed, particularly after extended surgical resections. This impairment is characterized by persistent reductions in fundamental voice frequency and dynamic vocal range.^4^ In addition, an increased roughness and hoarseness of the voice is frequently seen after CTR. Even though vocal impairment is usually mild to moderate and the improvement in overall quality of life is well documented, this reduction in voice quality is a common problem, especially in women.^5^

The functional impact of laryngotracheal surgery on the voice can be explained by the complex nature of the larynx, which combines breathing, vocalization, and swallowing. Laryngeal surgery results in permanent anatomic modification. In CTR, the cricoid arch is typically removed while the cricoid plate is preserved, which involves dissection of the cricothyroid ligament and loss of function of the cricothyroid joint. This joint centrally affects the tension of the vocal folds and thus is pivotal in adjusting the voice pitch.^6^

Here we present our preliminary experience in managing iSGS with a modified voice-sparing CTR technique in which technical refinements to the classic CTR allow preservation of the cricothyroid joint. We also analyze the impact of this modified voice-sparing CTR on functional outcomes.

## Methods

### Study Population

All patients with iSGS who underwent CTR between January 2017 and September 2022 at the Medical University of Vienna were included in this retrospective single-center analysis. The patients were grouped into standard CTR (Grillo/Pearson technique)^7^^,^^8^ or voice-sparing CTR. Medical records were analyzed to define patient characteristics and clinical variables, anamnesis, functional and endoscopic measurements, surgical interventions, and long-term outcomes. The study was approved by the Ethics Committee of the Medical University of Vienna (approval 2426/2020; June 22, 2020).

### Surgical Management

#### Standard CTR group

The patient was positioned with the neck hyperextended on the operating room table, and a laryngeal mask was placed.^9^ A cervical incision was performed above the cricoid, and the larynx and cervical trachea were dissected. Then the trachea was opened below the cricoid, and cross-table ventilation was initiated. All tracheal rings involved in the stenosis were removed. Then the cricoid arch was removed, and a dorsal mucosectomy of the cricoid plate was performed. A dorsal mucosal flap was generated from the distal trachea. A running polydioxanone suture (5.0 or 6.0) was placed to adapt the dorsal parts of the anastomosis and to cover the cricoid plate. The lateral and anterior portions of the anastomosis were performed with polydioxanone 4.0 single sutures. After a step-by-step adaptation of the anastomosis, all sutures were tied, and a final bronchoscopy was performed. In the absence of laryngeal edema, the laryngeal mask was removed at the end of the procedure. In the event of significant edema, a small (size 5 or 6) tracheostomy was placed below the anastomosis.

#### Modified voice-sparing CTR

**Figure 1:**
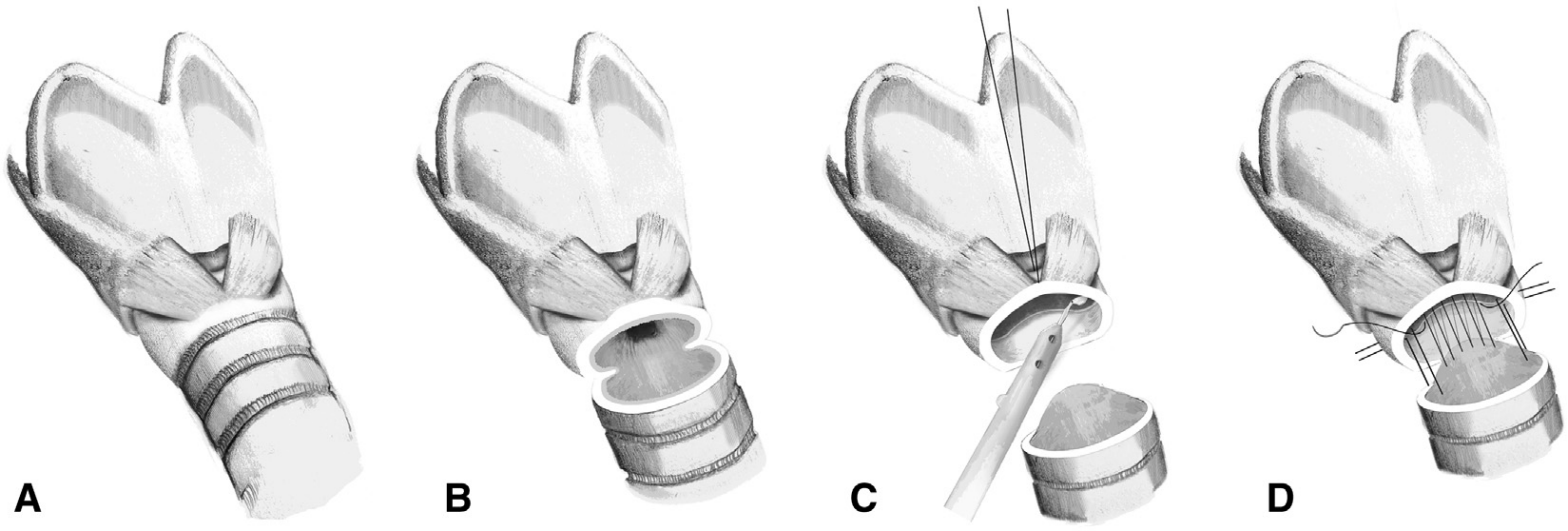
Surgical steps in the modified voice-sparing cricotracheal resection technique. A, Dissection of the larynx and cervical trachea. B, Preservation of the cricoid arch. C, Placement of a stay suture through the remaining cricoid arch and mucosectomy with a small beaver knife. D, Anastomosis with 2 lateral U stiches to slide the distal trachea into the subglottic airway.

The modified technique of voice-sparing CTR is characterized by preservation of the cricoid arch and thus the cricothyroid junction (Figure 1). After the airway distal of the cricoid arch was divided and all affected tracheal rings were removed, the mucosa behind the cricoid arch and the above the cricoid plate were assessed. If the arch was involved, a partial resection of the arch was performed by removing the lower half of it, with care taken to maintain the cricothyroid muscle. Then an anterior suture was placed through the remaining cricoid arch through which the subglottic airway could be raised for better exposure. A mucosectomy was performed with a small beaver knife, and all scar tissue was removed from the lateral and dorsal cricoid. The anastomosis was performed according to the standard CTR technique, but with the distal trachea intussuscepted into the subglottic airway with U stiches through the lateral cricoid on both sides.

### Functional Evaluation

According to our institutional standard, all patients with iSGS received a full functional and endoscopic evaluation before surgery and at 3 months postoperatively.^10^^,^^11^ A voice assessment was performed by experienced phoneticians, including measurements of voice pitch (in Hz), vocal range (in semitones) and fundamental voice volume (in dB; DiVAS software; XION GmbH). In addition, RBH grading—roughness, breathing and hoarseness of the voice, ranging from 0 (normal) to 3 (severe impairment)—along with the 9-point Voice Handicap Index (VHI) and phonation time were documented.^12^ Spirometry was used to evaluate respiratory function. In addition, patients were asked to self-assess their swallowing function using a structured form with scores ranging from 1 (no impairment) to 7 (severe dysphagia). A fiberoptic endoscopic evaluation of swallowing (FEES) for liquid, semisolid, and solid consistencies was conducted. Vocal fold function was assessed by chip on the tip endoscopy, and vocal fold movement, glottic closure, and level of vocalization (ie, true vocal fold, supraglottic phonation) were documented. All patients underwent bronchoscopy under general anesthesia before surgery to measure the extent and length of stenosis, distance to vocal fold level, and total length of the trachea. At 3 months after the surgery, another bronchoscopy was performed to assess healing of the anastomosis.

### Statistical Analysis

Statistical analyses was performed using SPSS 21 (IBM) and Prism 6 (GraphPad Software). The χ^2^ test and Fisher exact test were used to compare binominal variables, and the Student *t* test was used to compare variables in 2 independent groups. Values were recorded as mean ± SD or median and interquartile range. Categorical variables were recorded as number and percentage. All tests were 2-sided unless noted otherwise. A *P* value < .05 was considered to indicate statistical significance.

## Results

### Patient Characteristics

A total of 43 patients underwent CTR at the Medical University of Vienna between January 2017 and September 2022. These included 5 patients with a mean age of 57 years (range, 26-76 years) who were operated on between January 2022 and January 2023 using the voice-sparing CTR technique. All 5 of these patients were female, had an idiopathic etiology, and suffered from a Myer-Cotton grade III stenosis.^13^ One patient had undergone a singular endoscopic laser resection before CTR. The median length of stenosis was 15 mm (range, 10-35 mm), and the median total length of the trachea was 110 mm (range, 100-125 mm). Preoperatively, none of the patients had a tracheostomy, and none had any evidence of impaired vocal cord movement. Patient characteristics of both study groups are provided in Table 1.

**Table 1:** Patient characteristics and clinical variables in patients after voice-sparing and standard CTR

Variable	Total (N = 43)	Voice-sparing CTR (N = 5)	Standard CTR (N = 38)	*P* value
Sex, n (%)				.616
Male	5 (12)	-	5 (13)	
Female	38 (88)	5 (100)	33 (87)	
Age at surgery, y, median (range)	57.7 (26.3-77.1)	57.6 (26.9-76.2)	52.1 (26.3-77.1)	.403
Myer-Cotton grade, n (%)				.699
Grade I (0%-50%)	-	-	-	
Grade II (50%-70%)	4 (9)	-	4 (11)	
Grade III (71%-99%)	39 (91)	5 (100)	34 (89)	
Grade IV (100%)	-	-	-	
Length of stenosis, mm, median (range)	20 (5-45)	15 (10-35)	20 (5-45)	.256
Distance of stenosis to vocal cords, mm, median (range)	10 (0-30)	15 (10-20)	5 (0-30)	.027
Total length of trachea, mm, median (range)	120 (100-135)	110 (100-125)	125 (105-135)	.141
Etiology, n (%)				
Idiopathic	30 (70)	5 (100)	25 (66)	
Acquired	7 (16)	-	7 (18)	.228
Autoimmune	6 (14)	-	6 (16)	
Pretreatment				
Pretreated, n (%)	19 (44)	1 (20)	18 (47)	.362
Number of treatments, median (range)	3 (1-9)	1	3 (1-9)	.369
Tracheostomy at the time of referral, n (%)	1 (2)	-	1 (3)	.721
Comorbidities, n (%)				
Hypertension	10 (23)	1 (20)	9 (24)	.858
Diabetes mellitus	2 (5)	-	2 (5)	.609
Chronic obstructive pulmonary disease	2 (5)	-	2 (5)	.609
Hypothyroidism	10 (23)	3 (60)	7 (18)	.039
Hyperlipidemia	5 (12)	-	5 (13)	.400
Adipositas	2 (5)	-	2 (5)	.609
Gastroesophageal reflux	3 (7)	-	3 (8)	.526
Pulmonary embolism	1 (2)	-	1 (3)	.721
Asthma bronchiale	1 (2)	-	1 (3)	.721
Chronic renal insufficiency	1 (2)	-	1 (3)	.721

*CTR*, Cricotracheal resection.

### Surgical Treatment and Postoperative Outcomes

The mean operation time was 147 ± 47.4 minutes, with no significant differences between the voice-sparing and control groups. The average length of resection was 25 ± 8.2 mm. At the end of the procedure, the laryngeal mask could be removed in all patients; there was no evidence of glottic swelling in the voice-sparing group. Five patients (13%) in the control group required a utility tracheostomy, which could be removed at a median of postoperative day (POD) 5 (range, POD 2-9). In all 5 patients (100%), oral intake could be started on POD 1. The median length of hospital stay was 5 days (range, 4-7 days), with no significant difference between the 2 groups (*P* = .212). One patient in the voice-sparing CTR group developed a minor soft tissue emphysema after vomiting on POD 1, which was treated conservatively. In-hospital mortality and 30-day mortality were 0% in both the standard CTR and voice-sparing CTR groups. At the follow-up bronchoscopy 3 months after the operation, the majority of anastomoses had healed well; minor granulations had to be removed in 1 patient of the voice-sparing group. The mean total follow-up time was 10.1 ± 2.9 months. None of the patients required any reintervention or developed restenosis (Table 2).

**Table 2:** Surgical variables and outcomes in patients after voice-sparing and standard CTR

Variable	Total (N = 43)	Voice-sparing CTR (N = 5)	Standard CTR (N = 38)	*P* value
Operative time, min, mean ± SD	147 ± 47.4	114 ± 7.4		.063
Length of resection, mm, median (range)	25 (15-40)	25 (15-40)	25 (15-40)	.696
Postoperative utility tracheostomy, n (%)	5 (12)	-	5 (13)	.400
Time to decannulation, d, median (range)	5 (2-9)	-	5 (2-9)	
ICU stay, d, median (range)	1 (0-7)	0 (0-1)	1 (0-7)	.163
Hospital stay, d, median (range)	6 (3-16)	5 (4-7)	6 (3-16)	.212
Days to start of oral intake, median (range)	1 (1-5)	1	1 (1-5)	.192
Complications, n (%)				
Dehiscence	-	-	-	
Laryngeal edema	1 (2)	-	1 (3)	.721
Wound infection	-	-	-	
Soft tissue emphysema	1 (2)	1 (20)	-	.116
Pneumonia	-	-	-	
RLN paralysis	-	-	-	
In-hospital mortality	-	-	-	
30-d mortality	-	-	-	
Follow-up				
Follow-up time, mo, mean ± SD	42.4 ± 19.4	10.1 ± 2.9	46.6 ± 16.7	>.001
Restenosis, n (%)	-	-	-	
Granuloma, n (%)	1 (2)	1 (20)	-	.116
Lost to follow-up, n (%)	1 (2)	-	1 (3)	.721

*CTR*, Cricotracheal resection; *ICU*, intensive care unit; *RLN*, recurrent laryngeal nerve.

### Functional Outcomes

**Figure 2:**
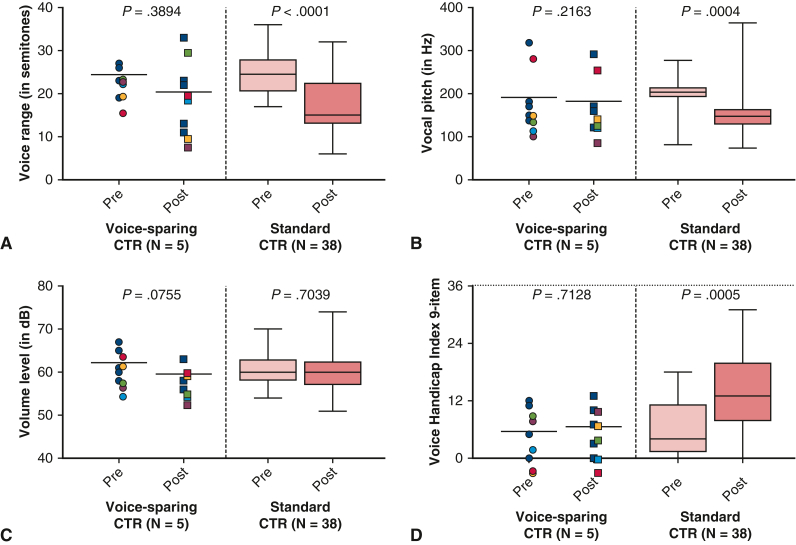
Preoperative and postoperative evaluation by voice-sparing cricotracheal resection (*CTR*) and standard CTR of voice range in semitones (A), vocal pitch (Hz) (B), volume level (dB) (C), and 9-item Voice Handicap Index (D). In the box-and- whisker plot, the *lower* and *upper* edges of the box represent the *lower* (25th percentile) and *upper* (75th percentile) quartiles. The *middle horizontal line* represents the median, and the *lower* and *upper* whiskers represent the minimum and maximum values of the nonoutliers. Additional dots represent outliers.

A complete functional follow-up was performed in all patients after voice-sparing CTR. Postoperative vocalization occurred at the level of the vocal folds in all patients (100%), and no changes in movement of the vocal folds were observed. No recurrent nerve palsy was documented postoperatively in any patient. At 3 months after voice-sparing CTR, evaluation of the RBH score showed no significant changes in roughness (*P* < 1.000), breathiness (*P* = .177), and hoarseness (*P* < 1.000) compared to the preoperative examination. In standard CTR, postoperative voice range was significantly lower compared to preoperative values (preoperative, 24.7 ± 4.7 semitones; postoperative, 17.2 ± 6.7 semitones; *P* < .001). Voice range was less impaired after voice-sparing CTR (preoperative, 24.4 ± 3.4 semitones; postoperative, 20.4 ± 8.8 semitones; *P* = .389) (Figure 2, *A*). In the standard CTR group, vocal pitch showed a reduction from an average of 199.2 ± 37.6 Hz to 150.0 ± 50.7 Hz (*P* < .001). In contrast, the voice-sparing CTR group showed only a minor, nonsignificant decrease from 191.0 ± 73.1 Hz to 181.8 ± 64.2 (*P* = .216) (Figure 2, *B*). Postoperatively, no changes in fundamental voice volume were observed in either study group (standard CTR group: 60.2 ± 3.9 dB vs 60.5 ± 3.9 dB [*P* = .337]; voice-sparing CTR group: 62.2 ± 3.7. dB vs 59.6 ± 3.2 dB [*P* = .075]) (Figure 2, *C*). On the 9-item VHI, in contrast to the standard CTR group (preoperative, 6.0 ± 5.5; postoperative, 13.8 ± 7.8; *P* < .001), no significant effect was observed in the voice-sparing CTR group (preoperative, 5.6 ± 5.7; postoperative, 6.6 ± 5.2; *P* < .712) (Figure 2, *D*).

**Figure 3:**
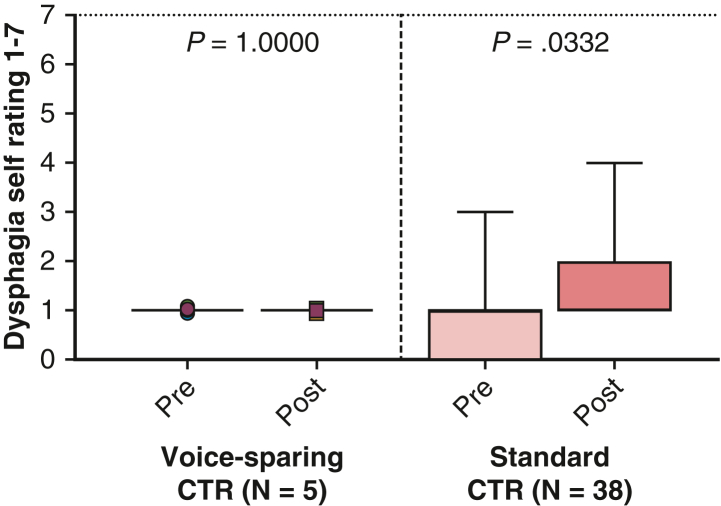
Preoperative and postoperative evaluation by voice-sparing cricotracheal resection (*CTR*) and standard CTR of dysphagia self-rating from 1 to 7. In the box-and-whisker plot, the *lower* and *upper* edges of the box represent the *lower* (25th percentile) and *upper* (75th percentile) quartiles. The *middle horizontal line* represents the median, and the *lower* and *upper* whiskers represent the minimum and maximum values of the nonoutliers. Additional dots represent outliers.

**Figure 4:**
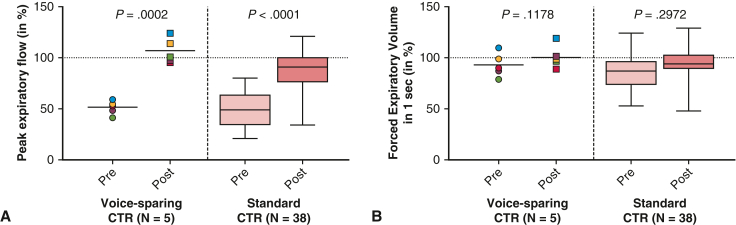
Preoperative and postoperative evaluation by voice-sparing cricotracheal resection (*CTR*) and standard CTR of peak expiratory flow (A) and forced expiratory volume in 1 second (B). In the box-and-whisker plot, the *lower* and *upper* edges of the box represent the *lower* (25th percentile) and *upper* (75th percentile) quartiles. The *middle horizontal line* represents the median, and the *lower* and *upper* whiskers represent the minimum and maximum values of the nonoutliers. Additional dots represent outliers.

All patients showed unremarkable deglutition and full oral intake early after surgery. FEES revealed no evidence of aspiration or penetration in any patient. The patients’ self-assessment of dysphagia from 1 to 7 showed no difference between the standard CTR and voice-sparing CTR groups (both 1.0 ± 0.0 vs 1.0 ± 0.0; *P* = 1.000) (Figure 3). Assessment of respiratory function showed a significant improvement in both groups. Mean peak expiratory flow increased significantly from 49.8 ± 16.3% preoperatively to 90.8 ± 19.4% postoperatively (*P* < .001) (Figure 4, *A*), and mean forced expiratory volume in 1 second improved from 87.1 ± 17.4% to 94.1 ± 15.2% (*P* < .072) (Figure 4, *B*) (Table 3).

**Table 3:** Functional outcome in patients after voice-sparing and standard CTR

Variable	Total (N = 43)		Voice-sparing CTR (N = 5)		Standard CTR (N = 38)	
	Value	*P* value	Value	*P* value	Value	*P* value
Phonation						
Preoperative level of phonation, n (%)						
Vocal cords	43 (100)		5 (100)		38 (100)	
Vestibular cords	-	1.000	-	1.000	-	1.000
Postoperative level of phonation, n (%)						
Vocal cords	43 (100)		5 (100)		38 (100)	
Vestibular cords	-		-			
Preoperative vocal cord movement, n (%)						
Unremarkable	39 (91)		5 (100)		34 (89)	
Reduced	4 (9)		-		4 (11)	
Immobile	-	.159	-	1.000	-	.160
Postoperative vocal cord movement, n (%)						
Unremarkable	41 (95)		5 (100)		36 (95)	
Reduced	2 (5)		-		2 (5)	
Immobile	-		-		-	
Preoperative glottic closure, n (%)						
Complete	42 (98)		5 (100)		37 (97)	
Incomplete	1 (2)	.159	-	1.000	1 (3)	.150
Postoperative glottic closure, n (%)						
Complete	40 (93)		5 (100)		35 (92)	
Incomplete	3 (7)		-		3 (8)	
RBH score						
Preoperative roughness, n (%)						
Grade 0	26 (61)		2 (40)		24 (63)	
Grade 1	15 (35)		3 (60)		12 (31)	
Grade 2	1 (2)		-		1 (3)	
Grade 3	-		-		-	
N/A	1 (2)	<.001	-	1.000	1 (3)	<.001
Postoperative roughness, n (%)						
Grade 0	9 (21)		2 (40)		7 (18)	
Grade 1	25 (58)		3 (60)		22 (58)	
Grade 2	5 (12)		-		5 (13)	
Grade 3	1 (2)		-		1 (3)	
N/A	3 (7)		-		3 (8)	
Preoperative breathiness, n (%)						
Grade 0	31 (72)		2 (40)		29 (76)	
Grade 1	11 (26)		3 (60)		8 (21)	
Grade 2	-		-		-	
Grade 3	-		-		-	
N/A	1 (2)	.017	-	.177	1 (3)	.004
Postoperative breathiness, n (%)						
Grade 0	28 (65)		4 (80)		24 (63)	
Grade 1	9 (21)		1 (20)		8 (21)	
Grade 2	2 (5)		-		2 (5)	
Grade 3	1 (2)		-		1 (3)	
N/A	3 (7)		-		3 (8)	
Preoperative hoarseness, n (%)						
Grade 0	25 (58)		2 (40)		23 (60)	
Grade 1	16 (38)		3 (60)		13 (34)	
Grade 2	1 (2)		-		1 (3)	
Grade 3	-		-		-	
N/A	1 (2)		-		1 (3)	
Postoperative hoarseness, n (%)		<.001		1.000		<.001
Grade 0	8 (19)		2 (40)		6 (16)	
Grade 1	26 (60)		3 (60)		23 (60)	
Grade 2	5 (12)		-		5 (13)	
Grade 3	1 (2)		-		1 (3)	
N/A	3 (7)		-		3 (8)	
Preoperative voice range (semitones), mean ± SD	24.7 ± 4.5	<.001	24.4 ± 3.4	.389	24.7 ± 4.7	<.001
Postoperative voice range (semitones), mean ± SD	17.7 ± 7.0		20.4 ± 8.8		17.2 ± 6.7	
Preoperative vocal pitch, Hz, mean ± SD	198.0 ± 43.2	<.001	191.0 ± 73.1	.216	199.2 ± 37.6	<.001
Postoperative vocal pitch, Hz, mean ± SD	154.7 ± 53.1		181.8 ± 64.2		150.0 ± 50.7	
Preoperative volume level, dB, mean ± SD	60.5 ± 3.9	.337	62.2 ± 3.7	.075	60.2 ± 3.9	.703
Postoperative volume level, dB, mean ± SD	60.0 ± 4.7		59.6 ± 3.2		60.1 ± 5.0	
Preoperative 9 Voice Handicap Index, mean ± SD	6.0 ± 5.5	<.001	5.6 ± 5.7	.712	6.0 ± 5.5	<.001
Postoperative 9 Voice Handicap Index, mean ± SD	12.9 ± 7.8		6.6 ± 5.2		13.8 ± 7.8	
Preoperative phonation time, s, mean ± SD	17.3 ± 6.6	.002	18.8 ± 8.3	.830	17.0 ± 6.4	.012
Postoperative phonation time, s, mean ± SD	20.0 ± 7.8		21.2 ± 10.8		19.8 ± 7.5	
Swallowing						
Preoperative swallowing, n (%)						
Unremarkable	42 (98)		5 (100)		37 (97)	
Aspiration	-		-		-	
Penetration	-		-		-	
N/A	1 (2)	1.000	-	1.000	1 (3)	1.000
Postoperative swallowing, n (%)						
Unremarkable	40 (93)		5 (100)		35 (91)	
Aspiration	-		-		-	
Penetration	-		-		-	
N/A	3 (7)		-		3 (8)	
Preoperative dysphagia self-rating (1-7), mean ± SD	1.2 ± 0.5	.033	1.0 ± 0.0	1.000	1.2 ± 0.5	.033
Postoperative dysphagia self-rating (1-7), mean ± SD	1.4 ± 0.8		1.0 ± 0.0		1.4 ± 0.8	
Spirometry						
Preoperative PEF, %, mean ± SD	49.8 ± 16.3	<.001	51.6 ± 6.9	<.001	49.5 ± 17.3	<.001
Postoperative PEF, %, mean ± SD	90.8 ± 19.4		107.0 ± 11.7		88.0 ± 19.2	
Preoperative FEV1, %, mean ± SD	87.1 ± 17.4	.072	93.0 ± 11.9	.117	86.1 ± 23.2	.297
Postoperative FEV1, %, mean ± SD	4.1 ± 15.2		100.4 ± 11.1		93.0 ± 15.6	

*CTR*, Cricotracheal resection; *RBH*, roughness-breathiness-hoarseness; *N/A*, not available; *PEF*, peak expiratory flow; *FEV1*, forced expiratory volume in 1 second.

## Discussion

To the best of our knowledge, this work is the first published description of a full voice-sparing CTR technique. We present surgical as well as functional results of an initial series of 5 patients treated with the modified CTR procedure and compare functional outcomes with a reference group of conventional CTR recipients.

Outcomes after standard CTR are well described. It was shown that in experienced centers, durable airway restoration can be achieved, but at the price of mild functional impairment of laryngeal functions. Although swallowing functions remain largely unchanged, alterations in voice quality are almost always seen. Patients after CTR usually have a decrease in their fundamental voice pitch, as well as their dynamic voice range.^4^^,^^14^ In the literature, resections in which the cricoid arch is removed are associated with a 30- to 40-Hz reduction in fundamental vocal frequency.^15^ These reports are in line with the mean decrease of 49 Hz in our reference group after standard CTR. In addition, there may be increases in voice roughness as well as hoarseness. These vocal changes can be explained by the modified functional anatomy of the larynx. A significant factor appears to be disruption of the cricothyroid junction by removal of the anterior cricoid arch during standard CTR. The cricothyroid joint regulates the tension of the vocal cords and consequently has a significant impact on vocal function.

Given the lack of alternatives, patients have previously accepted this moderate loss of voice quality in favor of airway restoration and a significant increase in overall quality of life.^16^ The restored respiratory function seems to outweigh the voice changes. Although experienced airway surgeons have speculated about modifications to the standard technique of CTR, the surgical challenges associated with preserving the cricothyroid joint have not yet been overcome. The main problem lies in the fact that surgeons need to perform an endoluminal mucosectomy and then slide the distal trachea into the subglottic airway. We have found 3 technical adaptations to be important: (1) lifting the subglottic airway with a midline stich around the cricoid arch to better expose the subglottic airway, (2) using a small beaver knife for the mucosectomy (because iSGS is characterized by a well-preserved layer between the mucosa and the perichondrium, mucosectomy with a knife is feasible); and (3) using U stiches for the 2 most lateral sutures, which helps slide the distal trachea into the subglottis.

Similar concepts of voice-preserving CTR were previously published by Tanner and colleagues.^17^ In their series, the cricothyroid muscle was scratched from the surface of the cricoid arch and thus preserved, the arch was removed, and cricothyroid anastomosis was performed. At the end, both cricothyroid muscles were reinserted into the anterior aspect of the trachea, distal to the anastomosis. With this adaptation, a joint-like function was recreated, which resulted in a smaller decrease in fundamental voice pitch (from 215 Hz to 201 Hz) after surgery. However, the VHI still decreased from 41 to 25. A comparison of the 11 patients reported by Tanner and colleagues and our present cohort favors the voice-sparing technique described herein, based on the full preservation of the cricothyroid joint with our technique as opposed to the creation of a new joint-like function of the cervical trachea.

However, a precondition for our voice-sparing CTR technique is that the subglottic stenosis is localized mainly on the posteriorly/laterally, as an anterior mucosectomy is most difficult. Therefore, careful selection of patients who are suitable for this intervention is crucial. Interestingly, age does not seem to influence the outcome of our modified technique. Our limited patient cohort ranged in age from 26 to 76 years, and all patients could be treated with a similarly good functional as well as surgical outcomes.

Along with the preservation of functional parameters, another main focus of airway surgery is on the long-term sustainability of the results. The efficacy and long-term outcomes after primary surgical resection for benign subglottic stenosis is now well demonstrated, and restenosis rates in experienced high-volume centers are extremely low. The radical nature of the resection of all affected tissue is a key factor for success. During our median follow-up of 10.1 months, no patient presented with restenosis. However, longer observation periods are needed to verify the equivalence to standard CTR in terms of sustainability.

This study has several limitations. Although the initial results are encouraging, the number of patients treated is still low, and the follow-up period is quite short. In addition, accurate selection of patients who can be offered voice-sparing CTR is important. In our study cohort, only 1 patient had a previous endoscopic pretreatment before voice-sparing CTR. This indicates that mainly patients with localized and therapy-naive stenosis can be selected for this procedure. In cases of extended and repeatedly pretreated stenoses, no risk should be accepted, and radical resection of the scar tissue is necessary.

In conclusion, the results of our voice-sparing CTR technique are encouraging. Patients seem to have an almost unchanged voice postoperatively, with simultaneous recovery of respiratory function and complete preservation of swallowing function.

## Conflict of Interest Statement

The authors reported no conflicts of interest.

The *Journal* policy requires editors and reviewers to disclose conflicts of interest and to decline handling or reviewing manuscripts for which they may have a conflict of interest. The editors and reviewers of this article have no conflicts of interest.
